# *Phellinus linteus* polysaccharide extracts increase the mitochondrial membrane potential and cause apoptotic death of THP-1 monocytes

**DOI:** 10.1186/1749-8546-8-25

**Published:** 2013-12-18

**Authors:** Leo JLD van Griensven, Harrie A Verhoeven

**Affiliations:** 1 Department of Bioscience, Plant Research International, Wageningen University and Research Centre, Droevendaalsesteeg 1, Wageningen 6700AA, The Netherlands

## Abstract

**Background:**

The differentiation resp. death of human monocytic THP-1 cells induced by polysaccharide extracts of the medicinal mushrooms *Phellinus linteus*, *Agaricus bisporus* and *Agaricus brasiliensis* have been studied. This study aims to identify leads for the causal effects of these mushroom components on cell differentiation and death.

**Methods:**

THP-1 cells were treated with different polysaccharide extracts of mushrooms and controls. Morphological effects were observed by light microscopy. Flow cytometry was applied to follow the cell differentiation by cell cycle shifts after staining with propidium iodide, changes of mitochondrial membrane potential after incubation with JC-1, and occurrence of intracellular reactive oxygen species after incubation with hydroethidine. Principal component analysis of the data was performed to evaluate the cellular effects of the different treatments.

**Results:**

*P. linteus* polysaccharide extracts induced dose-dependent apoptosis of THP-1 cells within 24 h, while *A. bisporus* and *A. brasiliensis* polysaccharide extracts caused differentiation into macrophages. A pure *P. linteus* polysaccharide had no effect. Apoptosis was inhibited by preincubating THP-1 cells with human serum. The principal component analysis revealed that *P. linteus, A. bisporus* and *A. brasiliensis* polysaccharide extracts increased reactive oxygen species production. Both *A. bisporus* and *A. brasiliensis* polysaccharide extracts decreased the mitochondrial membrane potential, while this was increased by *P. linteus* polysaccharide extracts.

**Conclusions:**

*P. linteus* polysaccharide extracts caused apoptosis of THP-1 monocytes while *A. bisporus* and *A. brasiliensis* polysaccharide extracts caused these cells to differentiate into macrophages. The protective effects of human serum suggested that *P. linteus* polysaccharide extract induced apoptosis by extrinsic pathway, *i.e.* by binding to the TRAIL receptor. The mitochondrial membrane potential together with reactive oxygen species seems to play an important role in cell differentiation and cell death.

## Background

*Phellinus linteus* is a basidiomyceteous mushroom that has been used for centuries as a traditional medicine in China, Korea and Japan for the treatment of various cancers and inflammation related diseases [1,2].

The medicinal effects of *P. linteus* have been attributed to β-glucans, phenols and terpenes [3,4]. The β-glucans are immunomodulators that can either enhance or attenuate the immune system of higher animals by inducing or inhibiting the production of pro- and anti-inflammatory cytokines [3]. The phenols, diterpenes and triterpenes had antioxidative effects and inhibited inflammatory mediators [5], which could prevent liver damage in rat and offer alternatives to the currently used antidiabetic agents [6]. *P. linteus* polysaccharide extracts are strongly antioxidative, and the purified polysaccharides comprise a mixture of 90% β-(1 → 3)(1 → 6) glucan and 10% α-glucan consisting of 85% glucose [7].

In experimental systems direct inhibitory effects of mushroom extracts on various tumors and tumor cell lines are caused by blockade of the cell cycle, apoptosis or both. Apoptosis is an essential process for tissue homeostasis, and its dysregulation might directly involve in initiation and progression of cancer [8]. Many studies have indicated that the upregulation of apoptosis or re-sensitization of cells to apoptotic stimuli showed promising potential for cancer therapy [9,10]. Apoptosis is initiated through extrinsic (death receptor mediated) and intrinsic (mitochondrion mediated) pathways [9]. Caspases, enzymes which belong to a growing family of cysteine proteases play an intermediary role. These enzymes are produced after death receptor binding (extrinsic pathway) and after cytochrome C release from damaged mitochondria (intrinsic pathway) [11].

A partially purified *P. linteus* polysaccharide extract significantly prolonged the survival rate of B16F10 melanoma implanted mice, inhibited tumor growth in NCI-H23 implanted nude mice, and reduced the pulmonary metastasis frequency of melanoma [12]. It could sensitize human prostate cancer cells to apoptosis both *in vitro*[13] and *in vivo* in nude mice [14]. Many cancer cell lines showed growth inhibition or apoptosis when treated by partially purified ethanol extracts of *P. linteus* and by the phenolic compounds hispolon or inotilone derived from these extracts [15]. Aqueous *P. linteus* extracts containing both water and ethanol soluble compounds induced cell cycle arrest [16], and apoptosis of cancer cells *in vitro* and *in vivo*[17-19].

Kim *et al.*[5] and Huang *et al.*[20] found that *P. linteus* organic extracts inhibited NF-κB, MMP-9 and MAPK activation *in vivo* and *in vitro*, leading to the conclusion that *P. linteus* may present a therapeutic approach for inflammation-associated disorders such as rheumatoid arthritis, psoriasis and other autoimmune afflictions. Shnyreva *et al.*[21] found that treatment of K562 cells with *P. linteus* ethanol extract increased the expression of the anti-inflammatory cytokine IL-10. Hispidin from *P. linteus* is a β-secretase inhibitor and is considered an anti-Alzheimer’s disease agent [22], while inotilone from *P. linteus* suppressed allergic inflammation in mice [20]. Suzuki *et al.*[23] demonstrated neuroprotective effects of a *P. linteus* culture filtrate in rats with ischemia induced cerebral infarction and Wang *et al.*[24] demonstrated curative effects of a *P. linteus* polysaccharide extract on thioacetamide-induced liver fibrosis.

We studied the differentiation of THP-1 cells, a human leukemia-derived monocytic cell line [25] after treatment with various extracts of *P. linteus*, *Agaricus bisporus* and *Agaricus brasiliensis* to assess the mechanisms underlying the *P. linteus* induced curative effects or alleviation of disease symptoms. Monocytes, dendritic cells and macrophages play important roles in host defense against infectious disease and cancer [26]. Activated macrophages produce cytokines and other inflammatory regulators such as COX-2, nitric oxide, and prostaglandins [27], leading to MAPK dependent signaling pathways and NF-κB dependent transcription regulation [28]. The purified *P. linteus* derived polysaccharides demonstrated the strong antioxidant effects and suppressed IFN-γ *in vitro*[7].

This study aims to investigate the effects of *P. linteus* polysaccharide extracts on the differentiation and death of THP-1 monocytes.

## Methods

### Chemicals, materials and equipment

All chemicals were obtained from Sigma-Aldrich, The Netherlands, unless otherwise mentioned. All materials and equipment was obtained from the Dutch supplier or their Dutch representative, unless otherwise stated.

### Fungal materials

Dried fruiting bodies of *A. bisporus* (Sylvan A15, *i.e*. strain Horst U, ATCC 62462) and of *A. brasiliensis* (Mycelia, Belgium, strain M7700) were obtained from the former Mushroom Experimental Station (Horst, The Netherlands), and wild *P. linteus* fruiting bodies were obtained from Amazing Grace Health Industries (Bangkok, Thailand) and had been identified by Dr. Usa Klinhom (University of Mahasarakham, Thailand). *P. linteus* samples were deposited in the collection of the Natural Medicinal Mushroom Museum of the Faculty of Biology of Mahasarakham University. *Cordyceps militaris* (L.) Link was donated by Dr. Jae-Mo Sung of Kangwon National University (Chuncheon, Korea). *Saccharomyces cerevisiae* (Bakers Yeast, Bruggeman, The Netherlands) was grown in malt extract medium with 2% glucose and collected by centrifugation.

### Cell and tissue culture

The human leukemia cell lines THP-1 (Cell Lines Service, Eppelheim, Germany) and K562 were donated by Prof. Huub F.J. Savelkoul, (Department of Cell Biology and Immunology, Wageningen UR) were grown in RPMI 1640 culture medium (Sigma-Aldrich, cat. R8758) supplemented with 10% heat-treated newborn calf serum (Gibco, cat. 161010–159) and penicillin/streptomycin (Sigma-Aldrich), at 37°C in 5% CO_2_ atmosphere in a humidified incubator. Human serum was purchased from Sanquin (Nijmegen, The Netherlands) and was a mixture of O, D(+) sera obtained by centrifugation of peripheral blood.

### Purification of *P. linteus, A. bisporus, A. brasiliensis* and *C. militaris* polysaccharides

Crude *P. linteus* extracts were obtained by fine grinding and sieving (size < 1 mm) of dry fruiting body. Then hot water extraction was applied by autoclavation for 20 min in excess water.

Partially purified polysaccharides were precipitated by addition of 2 volumes of 96% ethanol. Precipitate was dissolved in water, dialyzed, reprecipitated and characterized as described in Kozarski *et al.*[7].

Pure *P. linteus* polysaccharides were purified from dry powder that was initially extracted for16 h by ethanol at 65°C. The extracted tissues were collected by centrifugation in a Sorvall RC5B centrifuge at 7000 × *g* for 15 min and autoclaved twice in excess water (v/w ratio > 5). The mash was centrifuged in a Sorvall RC5B centrifuge at 7000 × *g* for 15 min and the resulting clear liquid was concentrated by evaporation in a Buchi R210 rotary evaporator at 45°C. After an initial precipitation by 2 volumes of cold ethanol the polysaccharides were suspended in water and subjected to a SEVAG treatment [29] with chloroform:butanol (6:1) to remove the bulk of the proteins. After reprecipitation with 2 volumes of ethanol, the partially pure *P. linteus* polysaccharides were suspended in cold 10% TCA to further remove protein remnants, and the liquid was clarified by centrifugation in a Sorvall RC5B centrifuge at 7000 × *g* for 15 min. Ethanol precipitation produced a white colored pellet of polysaccharide that, upon drying, was almost free of protein (< 0.1% w/w) and phenolic compounds (< 0.35% w/w) and was easily solved in water.

Ethanol extract was evaporated until dry and resuspended in ethanol. Insoluble components were removed by centrifugation in an Eppendorf F45 centrifuge at 10.000 × *g* for 10 min. All preparations were routinely quantified for polysaccharide content [30], phenol content by Folin-Ciocalteu assay [31], and protein content by Coomassie Brilliant Blue staining [32]. *P. linteus* polysaccharide extract was composed of approximately 90% β-(1 → 3)(1 → 6) glucan and 10% α-glucan and consisted of 85% glucose [7].

Polysaccharides were obtained by hot water extraction of *A. bisporus, A. brasiliensis,* and *C. militaris* powders as described before [7]. *A. bisporus* polysaccharide consists of glucose, galactose and mannose and contains (1 → 4)(1 → 6) α-glucan, (1 → 6)-β-glucan and mannogalactan; *A. brasiliensis* has a higher content of β-glucan, while *A. bisporus* has mannogalactan as its main polysaccharide [33].

### Scavenging activity

All polysaccharide extracts were investigated for their free radical scavenging activities by employing the stable free radical 2,2’-diphenyl-β-picrylhydrazyl (DPPH) (Sigma-Aldrich D-9132). DPPH was dissolved in 96% ethanol, and 1 mL was mixed with 0, 2, 5, 10, 20 and 50 μL of the extract in ethanol or water; appropriate standard ranges of gallic acid color controls were run in each series. Incubation was for 30 min at room temparature. Mixtures were centrifuged in an Eppendorf F45 centrifuge at 10.000 × *g* for 10 min. The absorbance of the reaction mixture was measured in quadruplicate at 485 nm in a 96 well plate using a Tecan (Switzerland) Sunrise 96 well microplate reader. The IC_50_ of the polysaccharides was determined and compared with the corresponding value for a concentration range of 0–200 μM Trolox. As a standard, the IC_50_ for Trolox in the DPPH assay was determined to be 35 μM.

### THP-1 differentiation and stimulation

The mature macrophage-like state was induced by treating THP-1 monocytes (500,000 cells/mL) for 1–2 days with 40 ng/mL phorbol 12-myristate 13-acetate (PMA; Sigma-Aldrich, cat.nr. 79352) in 24-well cell culture plates (Corning® Costar® from Sigma-Aldrich) with 1 mL cell suspension in each well. For the mushroom extracts, THP-1 cells were treated analogously. Cells received up to 1 mg of crude extract per mL and were daily checked for morphological changes under a Zeiss Axiovert 35 microscope. Cells were washed with phosphate buffered saline (PBS) (Sigma-Aldrich, cat.nr. T6146), fixated with 4% buffered formaldehyde and stained with 0.2% crystal violet (Sigma-Aldrich, cat.nr. V5265) in water to directly observe morphological changes and to quantitate the rate of differentiation. For the latter wells were shaken with 0.5 mL 96% ethanol and the absorption of the ethanol was measured at 590 nm.

### Trypan blue and fluorescent staining

Cell culture samples (15 μL) were taken from cell cultures and 1 volume of sterile filtered 0.4% trypan blue in PBS was added. After 3–4 min, cells were counted in a Fuchs-Rosenthal hemocytometer by a Zeiss Axioskop 40 microscope. For fluorescent staining, cells were washed with PBS and fixated in 4% buffered formaldehyde. Staining was performed by adding 10 μL of a mixture of 1% acridine orange (AO) (Sigma-Aldrich A9231) and 0.1% ethidium bromide (EB) (Sigma-Aldrich E 1510) in ethanol to 1 mL of cell suspension. Cells were observed in a Zeiss Axioskop, by excitation/emission wave lengths of 485 nm resp. 535 nm. As a rule experiments were repeated twice; for trypan blue 5 counts of at least 200 cells per point were carried out. For AO/EB staining a minimum of 500 cells was counted.

### Phagocytic activity

Green fluorescing polymer microspheres (0.01% (w/v)) of 1 μm size (G100, Duke Scientific Corp. USA) in PBS were opsonized by incubation with 10% (v/v) human serum for 1 h at 37°C. The particles were then washed with PBS. Phagocytosis was visualized by a Zeiss Axioskop after cells had been incubated for 16 h at 37°C with 10 μL microsphere suspension per mL cell culture.

### Flow cytometry

For cell cycle analysis using propidium iodide cells were collected from culture medium after 24 resp. 48 h cultivation by centrifugation in an Eppendorf F45 centrifuge at 80 × *g* for 10 min at 4°C and washed in cold PBS. Fixation was done with 70% ethanol for 30 min on ice; cells were then suspended in PBS and incubated with 40 μg/mL RNase A (Sigma-Aldrich, cat.nr. R6513) at room temperature for 1.5 h before starting cytometry, propidium iodide (PI) was added at 5 μg/mL. For ROS (reactive oxygen species) and MMP (mitochondrial membrane potential) measurements, experiments were performed in duplo, with one replicate stained with 2 μM JC-1 (Life Technologies Corporation cat. nr. T-3168)] and the other with 5 μM dihydroethidium (DHE, Sigma-Aldrich cat.nr. 37291).

A DAKO Cyan instrument (Beckman-Coulter Inc USA) was used for flowcytometric analysis, equipped with a 488 nm solid state blue green laser, operated at 10 mW output power. The standard filter/dichroic mirror set was used, and four fluorescence, forward scatter and sideward scatter signals were recorded, in both logarithmic and linear mode. Data were collected from the samples during a run of 30 s per sample. In this way, about 1500 to 6000 cells were analyzed during every run, allowing a comparison of cell growth or toxic effects of the treatments. Gating was applied in the forward/sideward scatter diagram to select for intact cells. Flow cytometry data were recorded in fcs3 file format and analysed using the free version of Cyflogic, version 1.2.1 (Cyflo Ltd, Finland). Selected data (fluorescence intensity, forward and sideward scatter) were exported to Excel for statistical analysis, using the StatistiXL version 1.8 (StatistiXl, Australia) plug-in.

Principal component analysis (PCA) was used to reveal complex interactions between various cellular properties to identify the discriminating parameters in the various treatments. The casewise PCA scores were plotted as round dots in graphs with the relevant PC’s on the horizontal and vertical axis. Component Score Coefficients (Eigenvectors) were indicated in these graphs with small squares as endpoint and identified by the parameters they are representing. For analysis of the cell cycle data (linear mode), signals were gated on pulse time to remove debris and cell aggregates. The gated fluorescence intensity histogram was divided into 4 regions, representing G1/G0, S and G2 phase of the cell cycle, with below G1 taken for induction of apoptosis (nuclear fragmentation) noise. Individual samples were normalized for cell number and cell number was added as an individual parameter to the PCA. Mitochondrial membrane polarization and ROS induction were cell parameters extracted respectively from JC-1 and DHE stained cells.

All data, except pulse duration, were recorded in logarithmic mode and represented as median value with CV, whereas pulse duration was represented as mean with CV. The entire data matrix was analyzed by PCA using StatistiXL v.1.8 (StatistiXl, Australia).

### Statisticsanalysis

Statistica for Windows - release 5.0 (StatSoft Inc., Tulsa, USA) was used for the analysis of variance (ANOVA). All experiments were at least twice repeated. Measurements within an experiment were done in triplicate or quadruplicate and data were expressed as mean ± standard deviation (SD). The experimental data were subjected to ANOVA and Fisher's LSD (least significant difference) test was performed to detect any significant difference (*P* < 0.05). Visual inspection showed dose-dependence after statistical preprocessing.

## Results and discussion

### THP-1 differentiation

**Figure 1:**
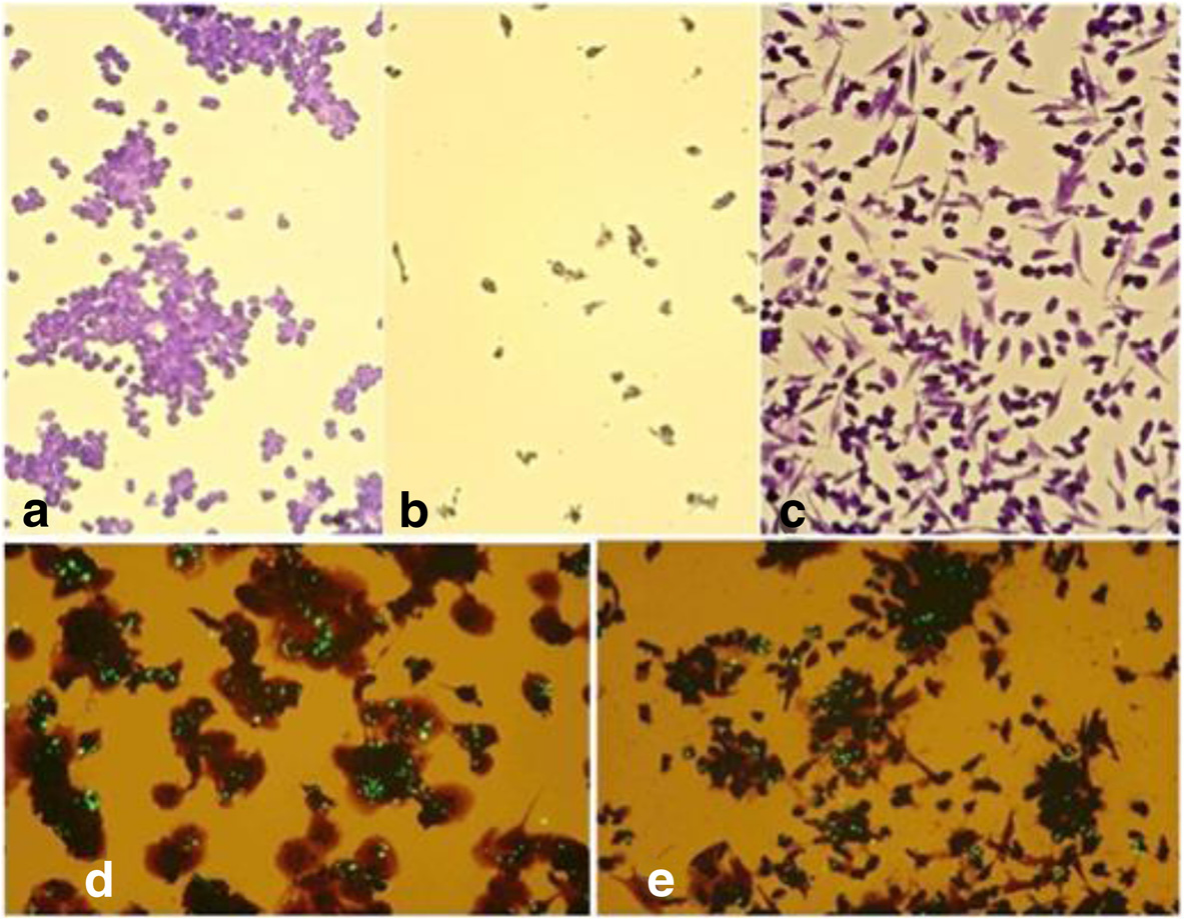
**THP-1 cells cultivated for 24 h with PMA (a), no addition (b) and 1 mg/mL *A. bisporus *polysaccharide extract (c); comparison of phagocytotic activity after PMA treatment (d) and *A. bisporus *polysaccharide extract treatment (e).** Cells were cultivated in a 24 wells plate, washed with PBS, fixated in 4% formaldehyde and stained by crystal violet. Figure 1**a** shows that PMA treated cells form aggregates after 24 h incubation and adhere to the plate. Figure 1**b** shows that non treated control cells do not adhere and are washed away. Figure 1**c** shows the effect of *A. bisporus* polysaccharide extract, stretching of the originally rounded cells, and adherence to the plate. Figure 1**d** shows the uptake of fluorescent latex particles in PMA treated THP-1 cells and Figure 1**e** shows uptake of particles in *A. bisporus* polysaccharide extract treated cells.

The effects of different polysaccharide extracts on the differentiation of monocytic THP-cells into macrophages were shown in Table 1. When THP-1 cells were incubated for 24 h with partially purified polysaccharide extracts of *A. bisporus* or *A. brasiliensis*, the cellular morphology changed from round cells growing in suspension to spindle shaped cells adhering to the wall of the culture plate (Figure 1c). This morphological change appeared analogous to the changes in THP-1 cells induced by phorbol 12-myristate 13-acetate (PMA), which converted THP-1 monocytes into macrophages [34]. In addition, the *A. bisporus* polysaccharide extract-treated THP-1 cells could absorb (fluorescent) latex particles of 1 μm diameter analogously to PMA activated THP-1 cells (Figure 1d and 1e). The *A. bisporus* and *A. brasiliensis* polysaccharide extracts induced differentiation within 24 h whereas that induced by PMA required at least 48–72 h (Table 1). The effects of a 24 h incubation with PMA on THP-1 cells were adherence, enlargement, and clustering. In the following 24–48 h, the cells differentiated into stretched spindle-shaped cells. A *C. militaris* polysaccharide extract caused initial wall adherence and spindle cell formation that quickly led to cell death. Laminarin, a linear β-(1 → 3)-glucan with β-(1 → 6) side chain branching on every tenth glucose unit along the polymer backbone [35], purified from the seaweed *Laminaria digitata,* had no differentiating effect, whereas zymosan, a β-(1 → 3)(1 → 6)-glucan rich protein carbohydrate complex from *Saccharomyces cerevisiae* had a limited differentiation-inducing effect (Table 1). The bacterial toxin lipopolysaccharide (LPS) as well as fixated *Escherichia coli* induced differentiation of THP-1 cells. Oat β-(1 → 3)(1 → 4)-glucan and *Saccharomyces sp*.-derived polysaccharides had no effects (Table 1). A possible explanation for the differentiation inducing activity of the *Agaricus* polysaccharide extracts is the presence of ergosterol derived 1α,25-dihydroxyvitamin D in the extracts [36,37]. Unlike *P. linteus, A. bisporus* and *A. brasiliensis* fruiting bodies contained large amounts of lipid-soluble ergosterol which can be metabolized into hydroxylated vitamin D2 and vitamin D4 [36,37]. The hydroxylated vitamin D3, a secosteroid of hydroxylated vitamin D2, binds to the vitamin D receptor on cells and plays a role not only in calcium uptake but also in differentiation [36,37]. Epidermal cells are an example; treatment with hydroxylated vitamin D3 improved calcium homeostasis and abolished proliferation [38]. Vitamin D3 and PMA increased macrophage differentiation of THP-1 cells, although vitamin D3 was less effective than PMA [34]. The morphological effects of PMA, *A. bisporus* and *A. brasiliensis* polysaccharide extracts on THP-1 cells were illustrated in Figure 1a and 1c, respectively.

**Table 1:** Effects of different extracts on differentiation of monocytic THP-1 cells into macrophages

**Species/Type**	**Macrophage-like morphology**	**Time required for changes (h)**	**Cytopathogenicity**
*A. bisporus*	++++	24-48	
*A. brasiliensis*	++++	24-48	
*C. militaris*	+++	24-72	After 24 h cell death
*P. linteus*	-	24	After 24 h apoptosis
*S. cerevisiae* (baker's yeast	-		
*Avena sativa* (Oat)	-		
PMA (40 ng/mL)	++++	48-96	
LPS (1 μg/mL)	++	72	
Laminarin	-		
Zymosan	+	48	
Fixated *E.coli (*5×10^8^/mL)	++	72	

THP-1 cells were cultivated in 24 well plates in RPMI 1640 complete medium to which polysaccharide extracts respectively activators had been added at 1 mg/mL, unless otherwise stated. After 24 h culture medium was decanted and cells were washed in PBS, fixated with 4% buffered formaldehyde and stained by 0.2% crystal violet as described in Methods: THP-1 differentiation and stimulation. Non-adhering cells as treated by *P. linteus* polysaccharide extract were collected and PBS washed by centrifuging at 80 × *g* for 5 min in an Eppendorf 45 centrifuge.

**Figure 2:**
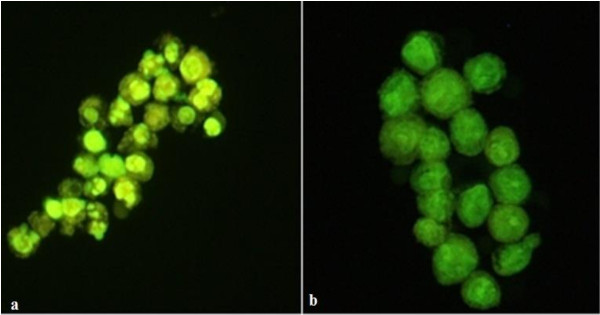
**Effect of partially purified *P. linteus *polysaccharide extract on the morphology of THP-1 cells. (a)** THP-1 cells cultivated with 1 mg/mL *P. linteus* polysaccharide extract for 24 h. Cells show strong shrinkage, nuclear condensation and nuclear fragmentation into apoptotic bodies. Cells were stained with an AO/EB mixture and immediately photographed using excitation/emission at 485/530 nm using a 20× objective. **(b)** Control THP-1 cells.

**Figure 3:**
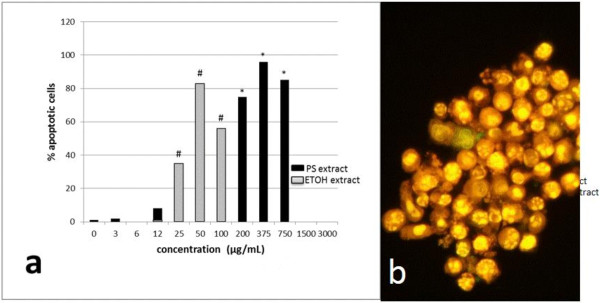
**Concentration-dependent induction of apoptosis in THP-1 cells.** Cells were cultivated for 24 h in the presence of different concentrations of partially purified *P. linteus* polysaccharide extract, respectively of ethanol extract of *P. linteus*. Cells were collected by centrifugation, fixated in 4% buffered formaldehyde and stained by AO/EB. A minimum of 500 cells was counted per point. Observation at excitation/emission at 485/530 nm, using a 20× objective. **(a)** Dose effect graph. **(b)** Microphoto of THP-1 cells treated by 375 μg *P. linteus* polysaccharide extract/mL medium. * and ^#^*P* < 0.05, significantly different from the previous values of the same concentration range.

**Figure 4:**
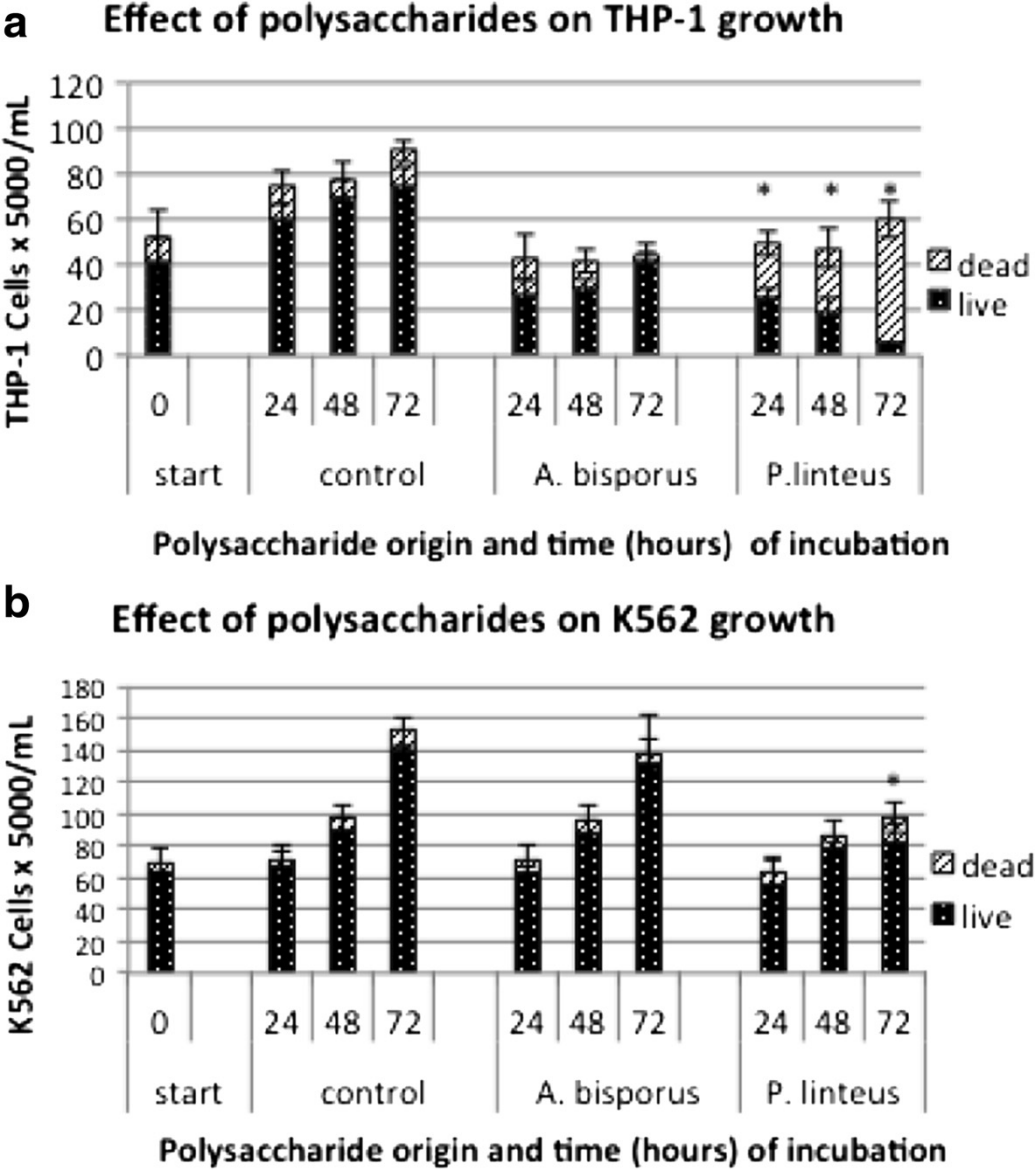
**Effect of *A. bisporus *polysaccharide extract and of *P. linteus *polysaccharide extract on the growth of THP-1 (a) and K562 cells (b).** Samples were taken at 24, 48 and 72 h after the start of incubation; counting was performed using trypan blue and non-colored cells were considered "live". Blue cells were considered "dead". A minimum of 500 cells cells was counted per point. *P < 0.01, significantly different from the values of the control group.

Contrary to the *A. bisporus-* and *A. brasiliensis-*induced effects, a partially purified *P. linteus* polysaccharide extract induced strong cell shrinkage, nuclear condensation, and fragmentation into apoptotic bodies after 24 h of exposure (Figure 2), leading to complete cell death within 48 h. The clear apoptotic volume decrease of the cells (Figure 2a) was caused by activation of K^+^ or Cl^-^ channels resulting in KCl efflux and exit of osmotically obliged water [39]. The induced apoptosis was polysaccharide extracts concentration-dependent and reached 100% of the cell population at 375 μg/mL after 24 h of incubation in the presence of *P. linteus* polysaccharide extracts (Figure 3). Student’s *t*-test showed that only the increase from 375 μg/mL to 750 μg/mL was the only one that showed no significant difference (*P* = 0.3) because the plateau value for the apoptotic percentage had been reached. In addition, the ethanol extract of *P. linteus* induced an analogous concentration-dependent apoptosis. At higher concentrations than the optimal values, most cells were decomposed into hardly recognizable remnants (Figure 3). The pure polysaccharide from *P. linteus* fruiting bodies contained less than 0.1% (w/w) protein as measured by the Bradford assay and less than 0.35% phenolic compounds (gallic acid equivalents) as measured by the Folin-Ciocalteu assay. This polysaccharide was free of DPPH-dependent scavenging activity. The pure *P. linteus* polysaccharide did not induce apoptosis in THP-1 cells, even at a concentration of 1 mg/mL culture medium. We incubated both THP-1 cells and human myeloid leukemia K562 cells with 1 mg/mL partially purified polysaccharide extracts for 24, 48, and 72 h, and determined the cell numbers and viability by the trypan blue exclusion assay to further test the cytopathic effect of *P. linteus* polysaccharide on cell growth (Figure 4). The *P. linteus* polysaccharide extracts caused significant inhibition of THP-1 cell growth after 24 h (*P* = 0.001) and 48 h (*P* = 0.007) and complete cell death after 72 h. The human leukemia cell line K562 was identically incubated with the same extract samples. The *P. linteus* polysaccharide extracts led to significantly slower cell growth than the control cells only after 72 h (*P* = 0.001). No increase in apoptotic cell death was observed in K562 cells incubated with the *P. linteus* polysaccharide extracts, indicating a specific interaction between the *P. linteus* polysaccharide extracts and THP-1 cells, rather than a general toxicity.

**Figure 5:**
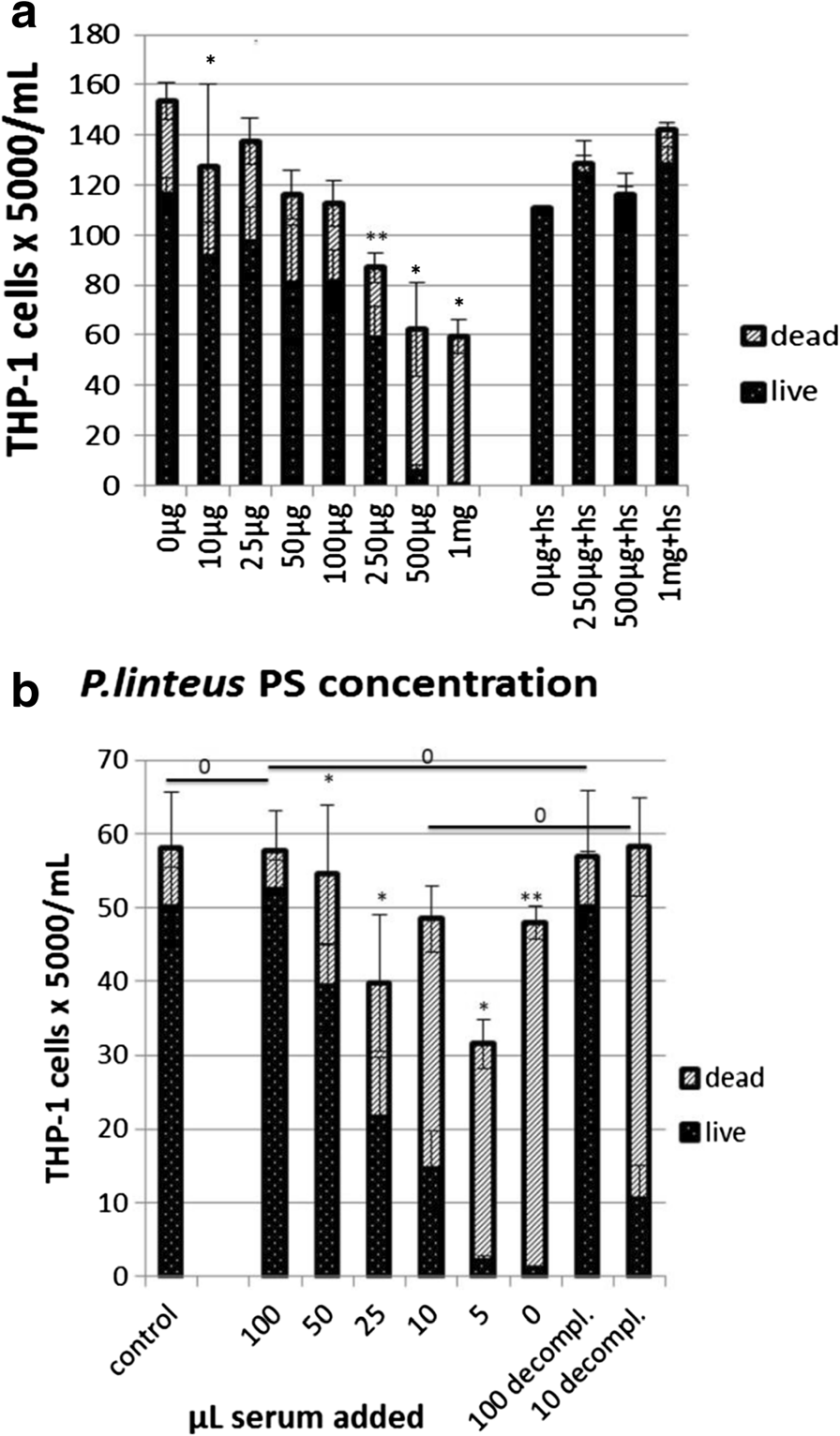
**Protective effect of human serum against *P. linteus *polysaccharide extract induced cell death in THP-1 cells (b) compared with the effects of varying *P. linteus *polysaccharide extract concentrations (a).** THP-1 cells were cultivated for 24 h in complete RPMI medium with 100 μg/mL *P. linteus* polysaccharide extract to which increasing amounts of human serum was added. Cells were stained with trypan blue and non-colored cells were considered "live". Blue cells were considered "dead". A minimum of 1000 cells was counted per point. Figure 5**a** shows concentration-dependent cytotoxicity of *P. linteus* polysaccharide extract. In the right section of the figure the effect is shown of simultaneous addition of 100 μL of human serum. Figure 5**b** shows the concentration-dependent inhibition of *P. linteus* polysaccharide extract cytotoxicity by human serum respectively by decomplemented serum. *P < 0.01, **P < 0.05, significantly different from the values of the previous (lower) concentration in the range; ^0^*P* > 0.05 not different between the concentrations indicated.

**Figure 6:**
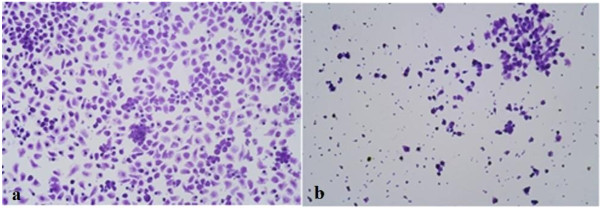
**Induction of differentiation in PBMCs by *P. linteus *polysaccharide extract. (a)** Freshly prepared PBMC's (10^7^ cells/mL) were incubated for 24 h in medium containing 1 mg/mL *P. linteus* polysaccharide extract. **(b)** Control, incubated in medium without *P. linteus* polysaccharide extract. After 24 h cultivation in a 24 well plate cells were drained,washed with PBS, fixated by 4% buffered formaldehyde and stained crystal violet.

Although both cell lines have a myeloid origin, THP-1 cells can differentiate into macrophages, while K562 differentiate into the erythroid direction. The cytopathogenicity for THP-1 cells did not change after opsonization of *P. linteus* polysaccharide extracts with excess complete human serum. However, when THP-1 cells were incubated with 1 mg/mL of *P. linteus* polysaccharide extracts and 5% (v/v) complete human serum, no cytopathic effect was observed: the THP-1 cells adhered to the wall of the culture plate and developed a phagocyte-like morphology. The same effect was observed when THP-1 cells were treated with the *P. linteus* polysaccharide extracts after preincubation of the cells for 6 h with complete human serum. The protection against *P. linteus*-induced cytotoxicity was dose-dependent in a range of 0.5-10% serum (Figure 5). Decomplementation of the human serum (heat treatment for 20 min at 58°C) had no effect on its protective activity. When freshly isolated human peripheral blood monocytes (PBMC’s) were treated with *P. linteus* polysaccharide extract, a considerable part of the monocyte population differentiated into wall adhering macrophage like cells within 24 h (Figure 6). The difference in effects between THP-1 cells and PBMC’s might be explained by the fact that the PBMC's were directly derived from a human serum protein environment, whereas the THP-1 cells had been cultured in calf serum for many generations leading to different receptor saturation. Freshly isolated PBMC's, contrary to-THP-1 cells, seemed to be naturally protected against the deleterious apoptosis-inducing effects of *P. linteus* polysaccharide extracts through binding of protective human serum components.

### Polysaccharide- or polyphenol-dependent apoptosis

As indicated above, both the partially purified polysaccharide extracts and the ethanol extract of *P. linteus* strongly induced apoptosis. When THP-1 cells were incubated for 48 h in the presence of 2–1000 μg/mL purified *P. linteus* polysaccharide, the MTT assay showed no metabolic effects. Microscopic observation employing acridine orange/ethidium bromide AO/EB staining and cell counting revealed that no apoptotic cells were present above the background level (0.1-0.3%). We had found before that *P. linteus* ethanol extract caused maximum expression of the apoptosis related genes Bcl-2 and Casp-9 in K562 cells [21]. Together this suggested that the polyphenols rather than the polysaccharides cause the apoptotic effects.

Partially purified polysaccharides apparently caused apoptosis due to the content of contaminating polyphenols which had remained in these samples, as already suggested by Kozarski *et al.*[7]. These antioxidant properties were apparently not protective against apoptosis in THP-1 cells. K562 cells that had been cultivated under the same conditions as THP-1 hardly showed effects of *P. linteus* polysaccharide extract treatment. However, K562 cells carry the glutathione S-transferase P1-1 (GSTP1-1) gene, which conjugates glutathione to electrophilic compounds and exerts strong antioxidative as well as anti-inflammatory effects [40]. The question then becomes whether the apoptosis induction by *P. linteus* is suicide-receptor (TRAIL)- driven (extrinsic) or caused by dysfunctioning mitochondria through ROS -driven insults (intrinsic).

### Cell cycle analysis

**Figure 7:**
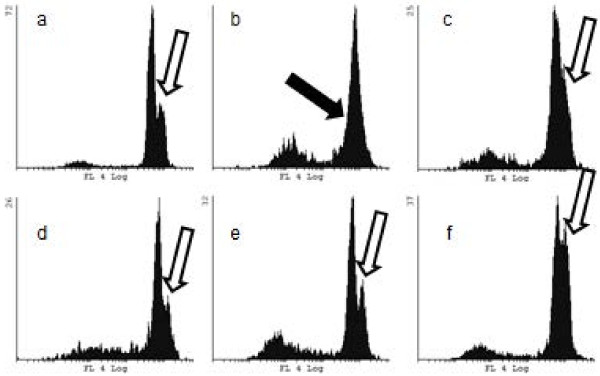
**FCM histograms of THP1 cells showing cellular DNA distributions after various treatments.** Shown are the histograms at 48 h of control **(a)**, *P. linteus* polysaccharide extract **(b)**, *P. linteus* polysaccharide extract plus HS **(c)**, PMA **(d)**, *A. bisporus* polysaccharide extract **(e)** and *A. brasiliensis* polysaccharide extract **(f)**. Fluorescence intensity of PI is presented on the logarithmic horizontal axis, cell count on the vertical axis. Histogram from cells is gated in FS and SS dotplot to exclude cell aggregates and cellular debris. Treatment with *P. linteus* polysaccharide extract results in cell cycle arrest in G1, with a skewed slope to sub G1 levels due to fragmenting nuclei and dead cells, indicated by the solid arrow in **(b)**. The cell cycle arrest is overcome by cotreatment with HS, resulting in a resumption of DNA synthesis, indicated by the open arrow in **(c)**. In the lower row, extracts from *A. bisporus* and *A. brasiliensis* result in comparable DNA profiles as PMA **(d)** with strong increases of the numbers of cells in the S-phase (open arrows).

Cells were analysed at 24 and 48 h after start of the treatments. The following treatments were tested: control, PMA, *A. bisporus* polysaccharide extract, *A. brasiliensis* polysaccharide extract, human serum, *P. linteus* polysaccharide extract, and *P. linteus* polysaccharide extract combined with human serum. After 24 h, the control cells contained a large percentage of S-phase cells, with a G1/G0 peak of low coefficient of variation (CV) (Figure 7). Treatment with *P. linteus* polysaccharide extracts strongly reduced the synthesizing cells (visible as accumulation of cells in S-phase), while treatment with human serum in combination with the *P. linteus* polysaccharide extract largely repaired the negative effect of the *P. linteus* polysaccharide extract. PMA treatment resulted in a comparable histogram to that obtained after incubation with the *A. bisporus* and *A. brasiliensis* polysaccharide extracts extracts (Figure 7d, e and f).

**Figure 8:**
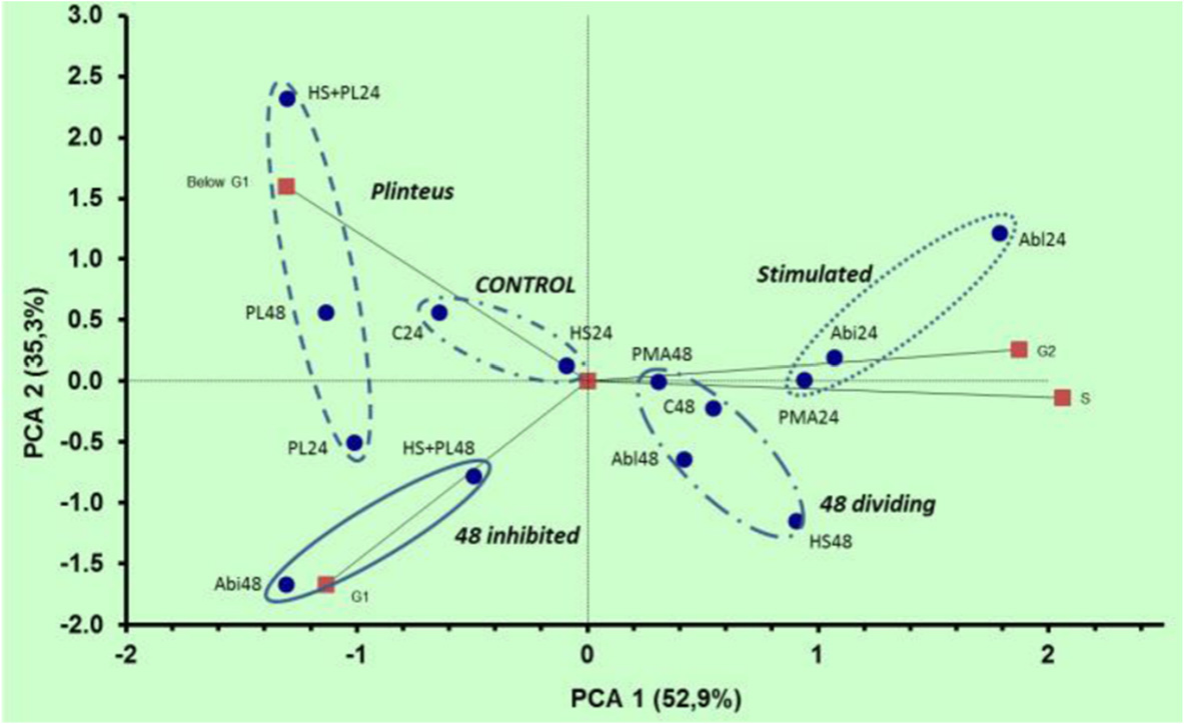
**PCA of the DNA flowcytometer data.** Data from FCM analysis of propidium iodide (PI) stained cells from each treatment were processed in Cyflogic FCM analysis package according to the following criteria: cells were gated on a window in the pulsetime PI intensity dot plot to exclude cell aggregates. The gated signals were represented as histograms: intensity, pulse time, forward scatter, sideward scatter. In the intensity plot, 4 windows were defined: DNA content below G1 phase of the cell cycle for apoptotic cells (Below G1), the window indicating G0/G1 phase (G1), a window for S-phase (S) and a window for G2/M phase (G2). In each window, the number of cells was expressed as % of total cell count in de gating window. The eigenvectors computed from these parameters are indicated on the plot by the small squares. Each dot is a separate treatment, indicated by the treatment code: C: control; Pl: *P. linteus* polysaccharide extract; Pl + HS: *P. linteus* polysaccharide extract complemented with human serum; HS: human serum; PMA: PMA treatment; Abi: *A. bisporus* polysaccharide extract; Abl: *A. brasiliensis* polysaccharide extract; codes are followed by the time at which cells were sampled (h) after begin of the treatment. A minimum of 1500 cells was counted in the gating window. Similar patterns are grouped and indicated with their most prominent feature in italics: Control for the control and HS24 treatment, Stimulated for the PMA and *Agaricus* polysaccharide extract treatments at 24 h, 48 dividing for PMA and *A. bisporus* and *A. brasiliensis* polysaccharide extracts treatments at 48 h, 48 inhibited for the cells arrested in G1 and *P. linteus* for the *P. linteus* polysaccharide extract treatments, resulting in sub G1 DNA levels due to fragmenting nuclei and dead cells.

In a separate experiment, cells were analysed for cell cycle progression after 24 and 48 h of treatment. PCA analysis of these DNA histograms is presented in Figure 8. The main difference in this PCA is the grouping of the individual treatments according to the duration of the treatment, except for *P. linteus* at 48 h. The main eigenvectors for this difference are G0/G1 transition to S/G2 for all treatments (eigenvectors G1, G2, S), except for *P. linteus* at 24 and 48 h. In the 24 h *P. linteus* polysaccharide extract-treated sample, a considerable contribution of apoptosis can be seen (eigenvector Below_G1) as nuclei below the G1 level, confirming the microscopic observations. After 48 h, all treatments exhibited a reduction of the number of cells in S and G2 phase through exhaustion of the culture medium.

The treatment combination of the *P. linteus* polysaccharide extract with human serum recovered the ability of cells to progress through the cell cycle both in the 24 and 48 h-treated samples, confirming the cytological observation with trypan blue exclusion assays. The 48 h *P. linteus* polysaccharide extract-treated sample showed increases in the mean pulse time (PT_mean, PT_CV), indication of the formation of cell aggregates, and in mean sideward scatter intensity (eigenvector SS_Mean), indicating increased cell density through pyknotic nuclei. Opsonization of the *P. linteus* polysaccharide extract with complete human serum had no effect on its apoptosis-inducing activity, but when the *P. linteus* polysaccharide extract was added to the THP-1 cells before or together with complete or heat-decomplemented human serum, no apoptosis was observed. Instead, the THP-1 cells showed rapid differentiation comparable to the effect of the *A. bisporus* polysaccharide extract and PMA. The inhibitory effect of human serum on *P. linteus* polysaccharide extract induced apoptosis suggested that human serum components might bind to the TRAIL receptor without causing harm. It also suggested that their binding was highly competitive to that of *P. linteus* components, thereby preventing the *P. linteus* polysaccharide extract from inducing apoptosis of THP-1 cells. Laminarin, a standard source of linear β-(1 → 3)-glucan with β-(1 → 6) side chain, had no effect on THP-1 cell differentiation, and zymosan also showed no effect either. Preincubation of THP-1 cells for 6 hours with 100 μg/mL DMSO-solubilized laminarin before the addition of mushroom polysaccharides extracts or PMA did not change the morphological response of the cells (Table 1).

The *P. linteus* polysaccharide extract seemed to bind to apoptosis-inducing death receptors like the TRAIL receptor on THP-1 cells, which is constitutively expressed in various tissues and upregulated during cell activation. If TRAIL ligands bind to the death receptors on the cell membrane, apoptosis can be induced in (most, but not all) tumor cells, but not in normal cells [41]. Human serum prevents this extrinsic apoptosis induction, presumably through the action of lysophosphatidic acid or sphingosine-1-phosphate resulting in inhibition of caspase-8 activation [42]. This renders our hypothesis on the saturation of TRAIL-like receptors by human serum components disputable.

The leukemia derived K562 cells are known to show resistance against TRAIL-induced apoptosis and do not respond to soluble factors that lead to apoptosis in other tumor cells [43]. K562 cells are high in glutathione (GSH) transferase and not very sensitive to ROS-induced apoptosis [44] contrary to THP-1 cells [45]. The strong antioxidant activity of crude *P. linteus* polysaccharides [7] could inhibit the possible intrinsic apoptosis pathway, which usually involves ROS generation and decrease of mitochondrial membrane polarity.

### Induction of intracellular ROS and changes in MMP

To further study the effects of *P. linteus* polysaccharide extract on THP-1 cells, we used the fluorescent dyes dihydroethidine (DHE) and JC-1 to quantify the amounts of ROS induced and the changes in mitochondrial membrane potential, respectively.

**Figure 9:**
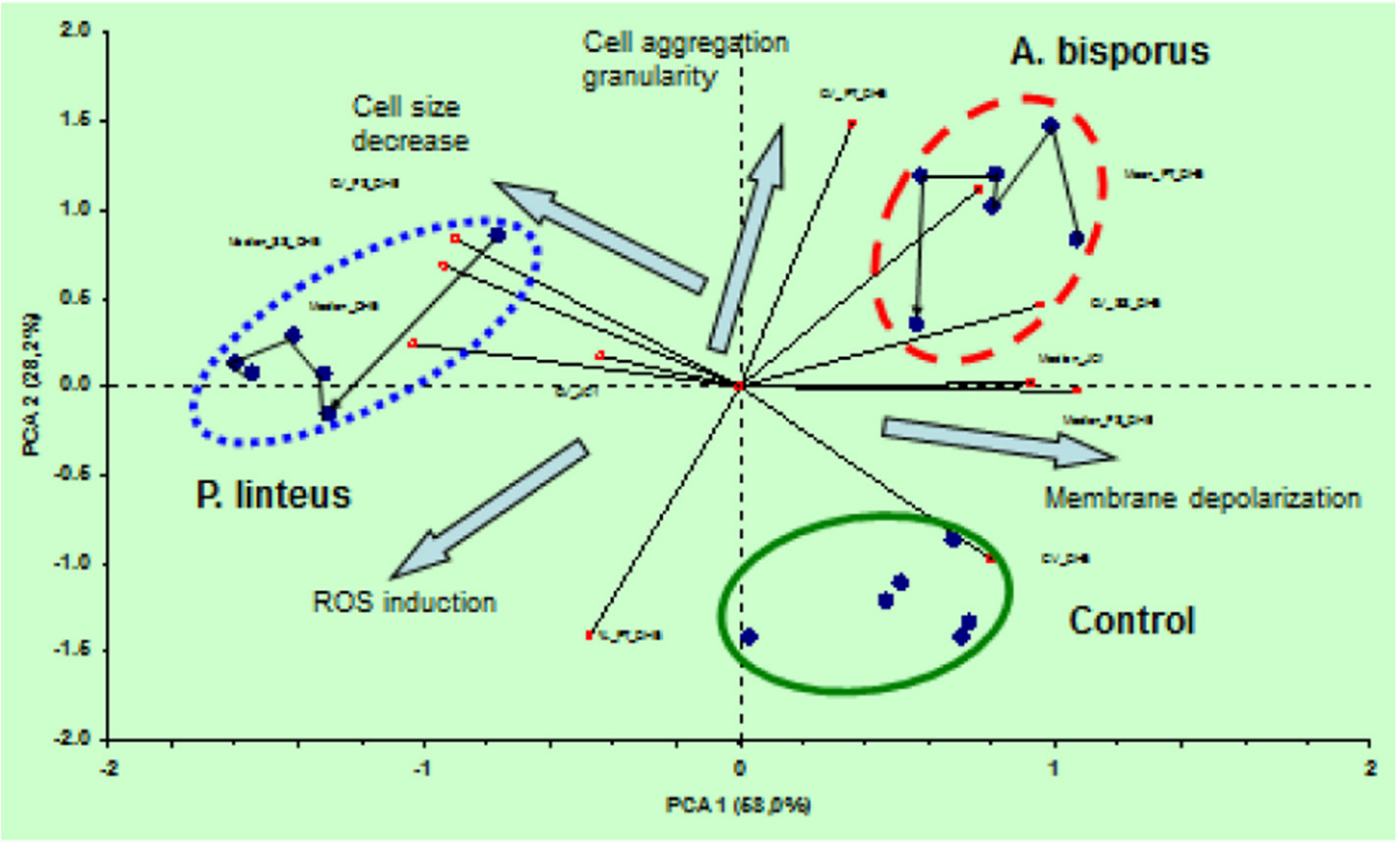
**PCA of DHE and JC-1 stained THP-1 cells.** Data from DHE and JC-1 stained cells were gated on forward and sideward scatter and processed by StatistiXl plug in for Excel. Selected parameters are: JC1 fluorescence intensity histogram: Median (Median_JC1), CV (CV_JC1); DHE fluorescence intensity histogram: median (Median_DHE), CV (CV_DHE); forward scatter on DHE signal histogram: median (FS_median_DHE), CV (CV_FS_DHE), sideward scatter: Median (Median_SS_DHE), CV (CV_SS_DHE); pulse time on DHE signal: mean (Mean_PT_DHE), CV (CV_PT_DHE) and% of total (%_PT_DHE). All eigenvectors computed from these parameters are indicated on the plot by the small squares, marked with the related vector code. The big arrows indicate the vectors for the observed cellular properties, extracted from the PCA. The marked populations comprise the different treatments, with each dot an individual treatment. Treatments are mentioned in the text, and are connected by the solid lines, the arrowheads indicate the increasing concentration of the extracts applied. A minimum of 3000 cells in the FS, SS gate was counted.

*P. linteus* polysaccharide extract gave rise to a concentration dependent increase of intracellular ROS, not much different of the effects of *A. bisporus* and *A. brasiliensis* polysaccharide extracts (Figure 9).

ROS are generated by mitochondrial processes, *i.e.*, by electrons liberated in the respiratory chain complexes I and III that react with electron acceptors and generate free radicals [46]. Oxidative stress occurs when ROS production is not balanced by intracellular antioxidants and antioxidant enzymes, and this may lead to mitochondrial dysfunctioning resulting in necrosis, apoptosis, and autophagy [46].

*A. bisporus*, *A. brasiliensis* and *P. linteus* polysaccharide extracts both equally increased ROS generation, providing another argument against ROS as the primary cause of the *P. linteus*-induced apoptosis in THP-1 cells (Figure 9). The *A. bisporus* polysaccharide extracts-treated cells showed a strong effect on aggregation, indicating attached cells by the Mean_PT_DHE and CV_PT_DHE eigenvectors, a hallmark of cell differentiation. The differentiation, *i.e.*, adherence to the wall of the cultivation flask, of THP-1 cells, was correlated with increased polarization of the plasma membrane potential [47] and Ca^2+^ influx [48]. The *A. bisporus* and *A. brasiliensis* polysaccharide extracts caused depolarization of the MMP while the *P. linteus* polysaccharide extract induced hyperpolarization in a concentration-dependent manner (Figure 9), suggesting that *A. bisporus* and *A. brasiliensis* polysaccharide extracts released mitochondrial Ca^2+^, thereby increasing cytoplasmic Ca^2+^ concentration, while *P. linteus* polysaccharide extract increased mitochondrial Ca^2+^ concentration, resulting in breakdown of the outer mitochondrial membrane and induction of apoptosis [48].

It seems there are two major factors determining differentiation and death of polysaccharide treated THP-1 cells, i.e. a death receptor (TRAIL-like) binding activity on the one hand, and oxidative stress and Ca^2+^ homeostasis on the other. The discovery that *bcl-2* modulates intracellular Ca^2+^ compartmentalization and that caspase cleavage of Ca^2+^ transporters might cause perturbation of intracellular Ca^2+^ homeostasis and affects the mode of cell death, provides support for the apoptosis- Ca^2+^ link [48]. The high scavenging activity of *P. linteus* polysaccharide extract, and even more so that of the ethanol extract, make the intrinsic apoptosis pathway rather unlikely as the primary cause of apoptosis in THP-1 cells. Taken together with the finding of concentration dependent increase of the MMP of THP-1 cells upon treatment with *P. linteus* polysaccharide extract and the inhibition of this effect by human serum, it is likely that the apoptosis of THP-1 cells induced by the *P. linteus* extract can only be caused by TRAIL receptor binding, thus triggering the mitochondrial pathway indirectly. Apoptosis through the mitochondrial pathway merely seems to be caused by disturbance of the mitochondrial Ca^2+^ homeostasis [49].

Changes in the MMP and Ca^2+^ homeostasis play a prominent role in the pathogenesis of age related loss of neuronal function, such as that occurring in Alzheimer's disease [50] and Parkinson's disease [51]. Hyperpolarizing compounds in *P. linteus* might restore Ca^2+^ homeostasis and thereby prevent the loss of neuronal function.

## Conclusion

*P. linteus* polysaccharide extracts caused apoptosis of THP-1 monocytes whereas these cells differentiated into macrophages in the presence of *A. bisporus* and of *A. brasiliensis* polysaccharide extracts. Human serum protected THP-1 cells against apoptosis by *P. linteus* polysaccharide extract. All polysaccharide extracts increased ROS in THP-1cells. *P. linteus* polysaccharide extract increased MMP, whereas *A. bisporus* and *A. brasiliensis* polysaccharide extracts decreased MMP. *P. linteus* derived compounds that strongly influence MMP and ROS may provide useful tools for the study of age related neurodegenerative diseases.

## Abbreviations

ANOVA: Analysis of variance; AO: Acridine orange; ATCC: American type culture collection; COX-2: Cyclooxygenase-2; CV: Coefficient of variance; DHE: Dihydroethidine; DPPH: 1, 1-diphenyl-2-picrylhydrazyl radical; EB: Ethidium bromide; FCM: Flow cytometry; HS: Human serum; IC50: 50% inhibitory concentration; IL-10: Interleukin-10; JC-1: Membrane permeant dye to measure mitochondrial membrane potential; LSD: Least significant difference; MAPK: Mitogen activated protein kinase; MMP: Mitochondrial membrane potential; MMP-9: Matrix metallopeptidase-9; MTT: Tetrazolium compound for measuring mitochondrial metabolic activity; NF-κB: Nuclear factor κB; OMM: Outer mitochondrial membrane; PBMC: Peripheral blood monocytic cell; PBS: Phosphate-buffered saline; PCA: Principal component analysis; PI: Propidium iodide; PMA: Phorbol myristate acetate; PS: Polysaccharide; ROS: Reactive oxygen species; TCA: Trichloacetic acid; TRAIL: Tumor necrosis factor related apoptosis inducing ligand.

## Competing interests

Amazing Grace Health Industries of Bangkok, Thailand, contributed to the costs of laboratory rental and materials of LJLDvG.

## Authors’ contributions

LJLDvG and HV conceived and designed the study. LJLDvG and HV performed experiments. HV interpreted the results. LJLDvG wrote the manuscript. Both authors read and approved the manuscript.
